# Investigation of antimicrobial susceptibility and virulence factor genes in *Trueperella pyogenes* isolated from clinical mastitis cases of dairy cows

**DOI:** 10.1002/fsn3.2431

**Published:** 2021-06-24

**Authors:** Iradj Ashrafi Tamai, Abdolmajid Mohammadzadeh, Taghi Zahraei Salehi, Pezhman Mahmoodi, Babak Pakbin

**Affiliations:** ^1^ Department of Pathobiology Faculty of Veterinary Science Bu‐Ali Sina University Hamedan Iran; ^2^ Department of Microbiology and Immunology Faculty of Veterinary Medicine University of Tehran Tehran Iran; ^3^ Medical Microbiology Research Center Qazvin University of Medical Sciences Qazvin Iran

**Keywords:** antimicrobial susceptibility, dairy cow, *Trueperella pyogenes*, virulence factor gene

## Abstract

*Trueperella pyogenes* is an opportunistic pathogen causing important diseases including mastitis and metritis in domestic animals such as dairy cows leading to prominent economic losses in food production industry. The aim of this study was to investigate bacterial species, antimicrobial susceptibility, and presence of virulence factor genes and genotyping of *T*. *pyogenes* isolates associated with summer mastitis cases from 22 different farms around Tehran, Iran. Fifty‐five percent of dairy cows with clinical mastitis symptoms was infected by *T*. *pyogenesis* indicated that this pathogen is the most important contributor to clinical mastitis in dairy cows in the present study. A significant correlation was illustrated between presence of virulence factor genes of isolated pathogen, biochemical patterns, and the utter infected types. Multidrug resistance susceptibility observed between isolates indicated the important need for prudent use of antimicrobials in treatment of mastitis caused by *T*. *pyogenes* and increased concerning of consumer health associated with recent problems of antimicrobial resistance. The categorization of isolates was implemented into seven different clonal related types by COX‐PCR at 80% of similarity cutoff with significance relationship to clonal types, CAMP test result and sampling time and biochemical profile. Regarding to the results obtained at the present study, *T*. *pyogenes* can be considered as an important typically cause of purulent and acute form of clinical bovine mastitis and loss of dairy productivity. Further studies with more sample size and high‐throughput omic methods in various sampling time and areas are suggested for study of this pathogen precisely.

## Graphical abstract

The aim of this study was to investigate bacterial species, antimicrobial susceptibility, presence of virulence factor genes, and genotyping of *T. pyogenes* strains associated with summer mastitis cases from 22 different farms around Tehran, Iran. Fifty‐five percent of dairy cows with clinical mastitis symptoms was infected by *T. pyogenesis* indicated that this pathogen is the most important contributor to clinical mastitis in dairy cows in the present study. Regarding to the results obtained at the present study, *T. pyogenes* can be considered as an important typically cause of purulent and acute form of clinical bovine mastitis and loss of dairy productivity.

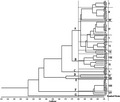

## 1 INTRODUCTION

*Trueperella pyogenes* is a Gram‐positive, nonsporeforming, nonmotile, opportunistic pathogen, short and rod‐shaped (coryneform) bacterium belonging to the Actinemycetales order. *T*. *pyogenes* was known previously as *Arcanobacterium pyogenes* and reclassified because of phospholipid composition, phylogenetic properties, and the presence of Vitamin K_2_. Growing condition of *T*. *pyogenes* is anaerobic; however, in a 7% CO_2_ atmosphere, it grows optimally (Pohl et al., 2018). It is widely considered ubiquitous, the inhabitant of skin, oropharynx, gastrointestinal, upper respiratory, and urogenital tracts of different domestic animal species. There are many suppurative infections caused by *T*. *pyogenes* including pneumonia in pigs; metritis, mastitis, liver abscesses, otitis, peritonitis, pyodermitis, endocarditis, osteomyelitis, lymphadenitis, and uterine diseases in cows and sheep, and abscesses in various wildlife species. As *T*. *pyogenes* is not a normal microflora in human and companion animals, infections caused by this bacterium are not common and occasionally associated with occupational exposures (Ribeiro et al., 2015). Several virulence factors, needed for pathogenesis of *T*. *pyogenesis*, existed in this pathogen and were reported by Machado and Bicalho (2014). *T*. *pyogenes* infection leading to tissue damage is associated with cholesterol‐dependent cytolysin, pyolisin encoded by *plo* gene, which is the primary virulence factor. Other important virulence factors are colonization and mucosal adherence involved in pathogenicity and considered for this bacterium which were described as fimbriae, collagen binding protein, and neuraminidases encoded with *fim(A*, *C*, *E and G)*, *cpbA*, and *nan(H and P)* genes, respectively (Machado & Bicalho, 2014).

Mastitis, one of the most important and costly diseases of dairy industry, is the inflammation of mammary glands. The major effects of this disease caused by mastitis pathogens include decrease in lactation and milk quality by enhancement of somatic cell count (SCC). Mastitis and the related diseases lead loss to producers approximately 2 billion dollars annually in the united states (Srednik et al., 2018). The pathogens causing mastitis include *Staphylococcus aureus*, *Streptococcus dysgalactiae*, *Trueprella pyogenes*, *Escherichia coli*, *Enterococcus spp*., *Bacillus spp*., and *Pasteurella haemolytica*. However; the major bacterial agents of mastitis are *S*. *aureus*, *E*. *coli*, and *T*. *Pyogenes*. No apparent change is visible in the udder in subclinical infections; however, decrease in milk production is occurred. On the other hand, clinical mastitis is known by swelling and pain in the udder with lactation decrease (Bogni et al., 2017). The most important factors which increase the risk of infection by *T*. *pyogenes* leading to metritis disease as a metabolic illness through the teat ends and vaginal canals is change in energy demand such as onset of lactation period resulted in increasing the risk of metabolic illness. Another factor has been determined for this disorder recently is change in feeding behavior (Pomeroy et al., 2017). Administration of antibiotics is the main choice for treatment of mastitis cases. However, overuse of antibiotics as treatment, preventive, and/or growth promoter agents in animal husbandry has led to the development of antimicrobial resistance in different bacterial species including *T*. *pyogenes*. Preventive activities and early treatment with correct antibiotic decrease the mentioned cost leading to higher economic proficiency in animal and food production at the cattle fields (Zhang, Zhao, et al., 2017).

Given the widespread use of antimicrobial agents, antimicrobial susceptibility tests along with molecular detection of antibiotic resistance genes are necessary after the diagnosis of the causative agent, to make the appropriate choice of therapy. Also, resistance to chloramphenicol, macrolides, and beta‐lactam antibiotics have been reported previously for *T*. *pyogenes* isolates (Machado et al., 2014). However, other treatments such as intravaginal immunization recently have been reported useful for prevention of urinary tract infection with whole‐cell vaccine or inactivated *T*. *pyogenes* pyolysin as an alternative treatment by antibiotics and preventive strategy for controlling the disease (Zhang, Wang, et al., 2017). The objectives of the present study were to investigate bacterial species associated with mastitis cases and subsequently determine the prevalence of *T*. *pyogenes* isolates involved in those infections. Furthermore, after genotyping of *T*. *pyogenes* isolates, the associations between detected clonal types, antimicrobial resistances, presence of virulence factors encoding genes, and persistence of infection were evaluated.

## 2 MATERIALS AND METHODS

### 2.1 Bacterial isolation and growth conditions

The study was conducted on 400 postpartum cows from 22 different farms around Tehran (range of number of cows was 8 to 14 cows per farm), which had clinical of summer mastitis. Clinical mastitis was diagnosed based on the presence of grossly altered secretion, systemic manifestations, and condition of the udder. Also, dairy cows with mastitis were treated by erythromycin, penicillin, kanamycin, neomycin, ceftriaxone, and tylosin antibiotics. Milk samples were collected over 9 months between April and December 2017 in individual sterile containers, stored in ice‐packed coolers, and sent to the bacteriology laboratory for further culturing and identification of potentially infectious agents. Smears were provided from all samples followed by bacterial cultivation on brain heart infusion agar (BHI) supplemented with 5% sheep blood and MacConkey agar (Merck, Germany), and incubation for 48 hr at 37℃ under aerobic and anaerobic conditions. Gram‐stained smears of all plated isolates were prepared. To identify *T*. *pyogenes* isolates, several biochemical tests were performed on the isolates (Malinowski et al., 2011). Isolated bacterial isolates other than *T*. *pyogenes* were also identified using morphological comparison and biochemical tests.

### 2.2 Antimicrobial susceptibility test

Resistance profiles of the isolated *T*. *pyogenes* isolates were determined on Mueller‐Hinton agar (Merck, Germany) supplemented with 5% sheep blood using the Kirby‐Bauer disk diffusion method according to the Clinical and Laboratory Standards Institute protocols (Weinstein et al., 2019). Twenty‐three antibacterial disks (MAST) were used, including gentamicin (GM 120 µg), ampicillin (AP 25 µg), penicillin G (PG 10 units), enrofloxacin (ENF 5 µg), tetracycline (T 30 µg), amoxicillin (A 25 µg), spectinomycin (SPC 25 µg), trimethoprim sulfamethoxazole (TS 25 µg), erythromycin (E 15 µg), ciprofloxacin (CIP 5 µg), cefalexin (CFX 30 µg), ceftriaxon (CRO 30 µg), cefepime (CPM 30 µg), cefixime (CFM 5 µg), ceftiofur (CEF 30 µg), azithromycin (ATH 15 µg), streptomycin (S 300 µg), lincomycin (L 2 µg), rifampicin (RP 5 µg), chloramphenicol (C 30 µg), minocycline (MN 30 µg), levofloxacin (LEV 5 µg), and tylosin (TY 30 µg). Results were recorded after 48–72 hr of incubation.

### 2.3 DNA extraction and polymerase chain reaction (PCR)

The isolated bacteria were cultured in TSB broth (Merck, Germany) supplemented with 5% bovine serum and incubated at 37 ℃ for 48 hr. In the next step, 3 ml of cultured TSB broth was centrifuged at 8,000 g for 10 min at 4 ℃, followed by washing the pellet once with saline solution. At last, genomic DNAs were extracted using a commercial DNA extraction kit for Gram‐positive bacteria according to the manufacturer's instruction (MBST, Iran). The extracted DNA samples were stored at −20℃ for further investigation. A PCR assay, which targeted a 16S‐23S rDNA intergenic spacer region in the genomic DNA, was used to confirm the identity of isolated *T*. *pyogenes* isolates.

PCR was conducted in 25 µl reaction mixtures containing 12.5 µl Taq DNA Polymerase 2x Master Mix Red (Cat No. A180306, AMPLIQON), 10 pmol of each primer (BIONEER, Korea) (F: 5'‐ GTT TTG CTT GTG ATC GTG GTG GTT ATG A‐3' and R: 5'‐AAG CAG GCC CAC GCG CAG G‐3'), and 2 µl of DNA template (Ülbegi‐Mohyla et al., 2010). The reactions were carried out in a thermocycler (TC‐512 Techne, England) as follows: an initial denaturation at 95℃ for 10 min, then 30 cycles of 95 ℃ for 30 s, 64℃ for 15 s, and 72 ℃ for 30 s, and a final extension at 72℃ for 7 min. The amplification products (5 µl) were resolved by electrophoresis on 1.5% agarose gel in 1X TBE buffer for 1 hr at 100 V. Afterward, the agarose gel was stained with 1 µg/ml ethidium bromide (CinnaGen, Iran) and screened using an UV‐Trans‐illuminator (BIORAD, UK). The positive control was *Trueperella pyogenes* isolate Arash114 (Accession Number CP028833). Negative controls consisting of the PCR mixture without addition of DNA templates were included in all PCR runs.

### 2.4 Screening of genes encoding putative virulence factors

**FIGURE 1:**
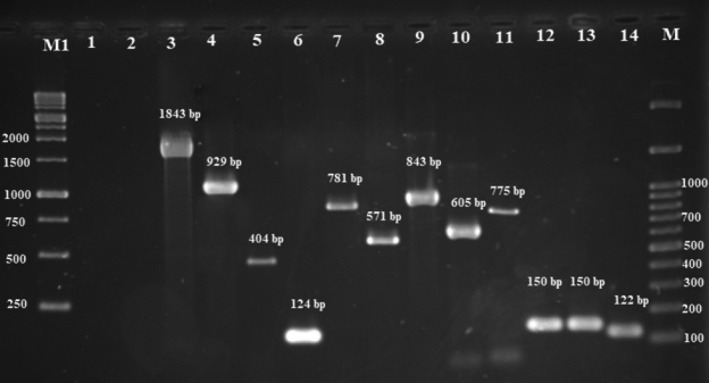
PCR analysis of genes encoding virulence factors. Line M1: Ladder 1kb, line 1and 2: Control negative, line 3: tetW, line 4: fimG, line 5: ermB, line 6: cbpA, line 7: nanH, line 8: ermX, line 9: fimC, line 10: fimA, line 11: fimE, line 12: nanP, line 13: plo, line 14: 16S‐23S, line M: Ladder 100 bp

**FIGURE 2:**
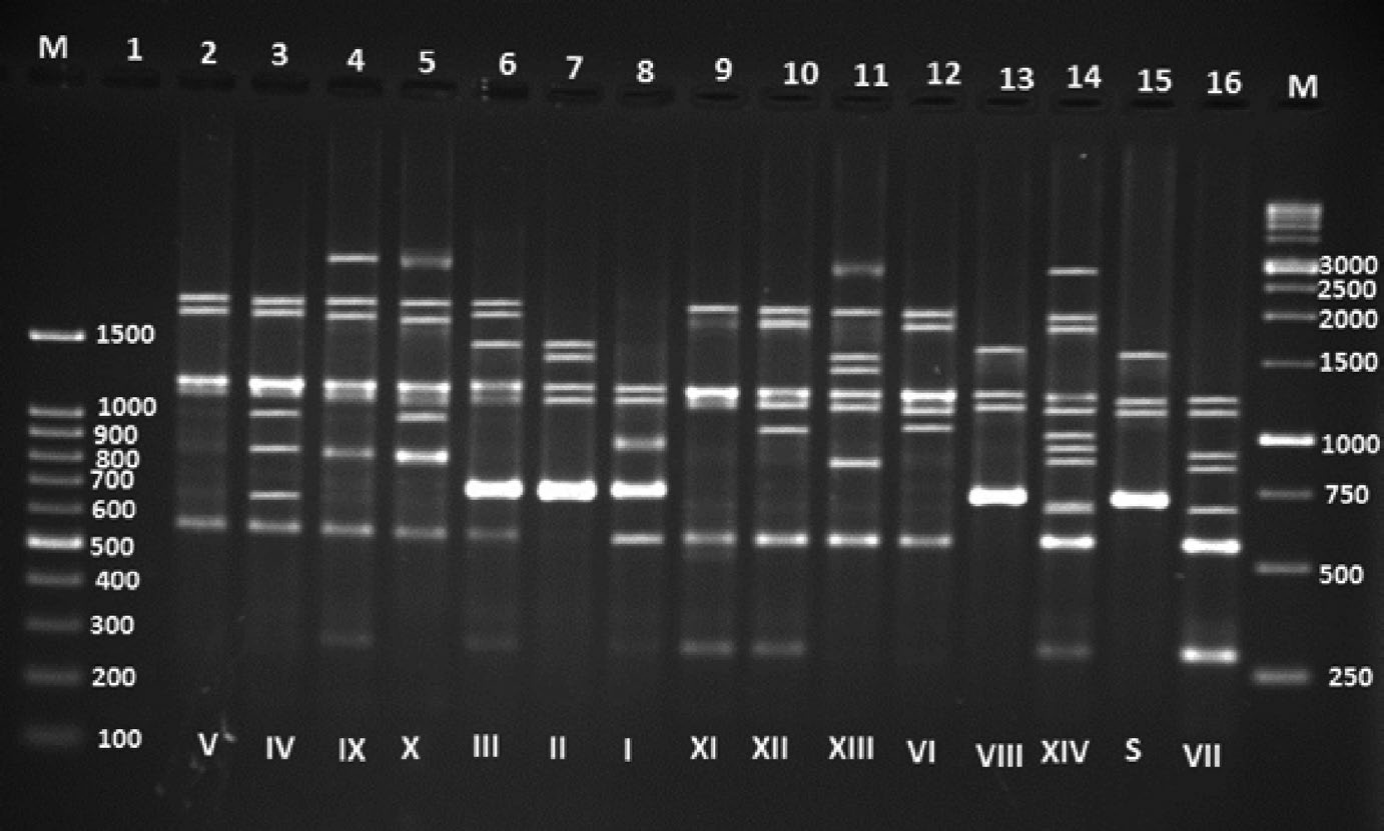
Example of DNA banding patterns obtained for T. pyogenes isolates by BOX‐PCR fingerprinting

Single PCR method was used to evaluate the presence of putative *T*. *pyogenes* virulence genes including *plo*, *nanH*, *nanP*, *cbpA*, *fimA*, *fimC*, *fimE*, and *fimG*, macrolide resistance genes *erm* (*X*) and *erm* (*B*), and tetracycline resistance gene *tet* (*W*) (Bicalho et al., 2012; Silva et al., 2008). PCR assays were performed in a reaction mixture with the final volume of 25 µl containing 10 pmol of each primer (BIONEER, Korea), 12.5 µl Taq DNA Polymerase 2x Master Mix Red (Cat No. A180306, AMPLIQON), and 2 µl of DNA template. Primer sequences and PCR conditions are listed in (Table 1). PCR reactions were performed in a TECHNE thermal cycler (TC‐512 England). Finally, PCR products were analyzed as previously mentioned. Positive controls including *plo* (MF458305), *nanP* (MF688763), *nanH* (MF688764), *fimA* (MF996852), *fimE* (MF155550), *fimC* (MF155551), *fimG* (MF978249), *cbpA* (MF688765), *ermX* (MF688766), *ermB* (MF688767), and *tetW* (MF996853) genes and correspond to code of the reference strain, *T*. *pyogenes* ATCC 19411, and negative controls consisting of the PCR mixture without addition of DNA templates were included in all PCR runs (Figures 1 and 2).

**TABLE 1:** Primer sequences and PCR conditions

Virulence factor / Target gene	Primer sequence (5'−3')	Product (bp)	Annealing (°C)
Pyolysin *(plo)*	F: TCATCAACAATCCACGAAGAG	150	60
	R: TTGCCTCCAGTTGACGCTTT		
Neuraminidase H *(nanH)*	F: CGCTAGTGCTGTAGCGTTGTTAAGT	781	60
	R: CCGAGGAGTTTTGACTGACTTTGT		
Neuraminidase P *(nanP)*	F: TTGAGCGTACGCAGCTCTTC	150	60
	R: CCACGAAATCGGCCTTATTG		
Collagen‐binding protein *(cbpA)*	F: GCAGGGTTGGTGAAAGAGTTTACT	124	60
	R: GCTTGATATAACCTTCAGAATTTGCA		
Type A fimbria *(fimA)*	F: CACTACGCTCACCATTCACAAG	605	57
	R: GCTGTAATCCGCTTTGTCTGTG		
Type G fimbria *(fimG)*	F: ACGCTTCAGAAGGTCACCAGG	929	57
	R: ATCTTGATCTGCCCCCATGCG		
Type E fimbria *(fimE)*	F: GCCCAGGACCGAGAGCCAGGGC	775	55
	R: GCCTTCACAAATAACAGCAACC		
Type C fimbria *(fimC)*	F: TGTCGAAGGTGACGTTCTTCG	843	60
	R: CAAGGTCACCGAGACTGCTGG		
Resistance to macrolide *(ermX)*	F: GTTGCGCTCTAACCGCTAAGGC	571/657	60
	R: CCATGGGGACCACTGAGCCGTC		
Resistance to macrolide *(ermB)*	F: GAAATTGGAACAGGTAAAGG	404	53
	R: TTTACTTTTGGTTTAGGATG		
Resistance to tetracycline *(tetW)*	F: GACAACGAGAACGGACACTATG	1843	55
	R: CGCAATAGCCAGCAATGAACGC		

### 2.5 Genotyping

**FIGURE 3:**
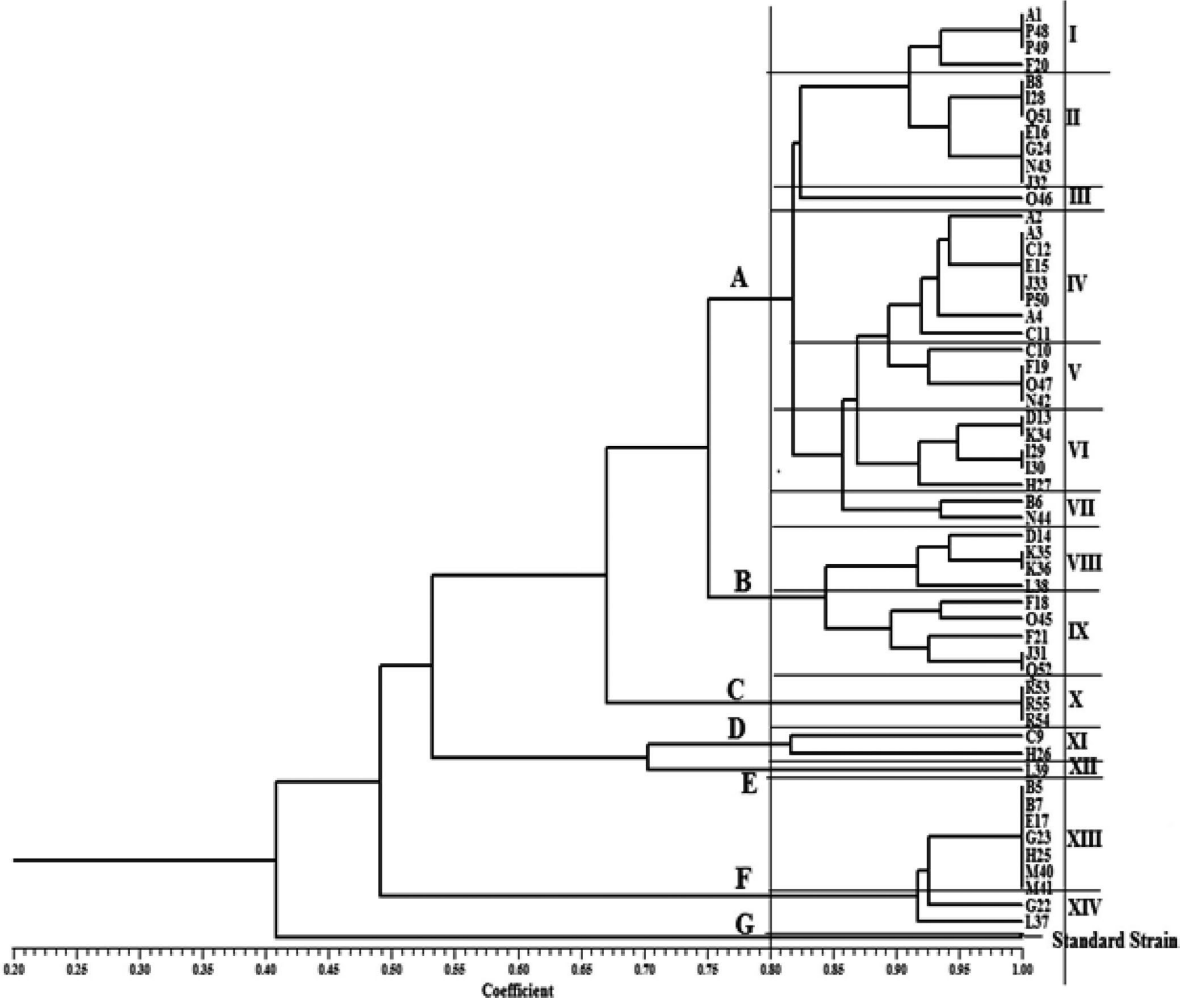
Dendrogram of BOX‐PCR patterns from the 55 isolates generated by NTsys software. The vertical line represents the similarity cutoff level of 80%

All isolates obtained from summer mastitis were evaluated with genomic DNA fingerprints generated by BOX‐PCR using BOXA1R (5'‐CTACGGCAAGGCGACGCTGACG‐3') primer (Silva et al., 2008). Genomic DNA of a reference strain of *T*. *pyogenes* (ATCC 19,411) and distilled water were used in BOX‐PCR as positive and negative controls, respectively. Reaction mixtures were prepared in a total volume of 25 µl containing a primer concentration of 2 µM (BIONEER, Korea), 12.5 µl Taq DNA Polymerase 2x Master Mix Red (Cat No. A180306, AMPLIQON), and 100 ng of template DNA. The reactions were carried out as follows: initial denaturation at 95℃ for 2 min, then 34 cycles of 95℃ for 1 min, 53℃ for 1 min, and 72℃ for 5 min, and a final extension step at 72℃ for 10 min. The amplification products (5 µl) were resolved by electrophoresis on 1.5% agarose gel for 3 hr at 70 V and visualized as above. The 1/0 (presence/absence) binary matrices were processed by NTsys program (NTSYSpc version 2.10e) to calculate the similarity coefficients and construct the clustering tree with an unweighted pair‐group method using arithmetic averages (UPGMA). The dendrogram was confirmed to be stable based on our repeated verifications (Figure 3). The similarity cutoff level to identify clonally related isolates was set up at 75% (Hadimli & Kav 2011; Silva et al., 2008).

### 2.6 Statistical analysis

The data were described by absolute and relative frequencies. Statistical analyses were done by SPSS software version 23.0 using chi‐square and Fisher's exact tests. The *p* value ≤.05 was considered to be statistically significant.

## 3 RESULTS AND DISCUSSION

### 3.1 Isolation and identification of *T. pyogenes*

Fifty‐five *T*. *pyogenes* isolates (13.75%) were isolated from 400 collected samples, 12 (21.82%) of which were isolated as pure cultures, while the other 43 isolates (78.18%) were obtained from mixed cultures. Other bacterial species which were isolated along with *T*. *pyogenes* are listed in (Table 2). Overall, *T*. *pyogenes* was the predominant bacterium isolated from the clinical samples. From 55 *T*. *pyogenes* isolates, 18, 28, and 9 isolates were from cows with mastitis symptoms during the spring, summer, and fall respectively. Small, irregular Gram‐positive coccobacilli were observed in smears obtained from the samples. After 48 hr, small white colonies with β hemolysis were detected on the blood agar plates, but no growth was observed on the MacConkey agar plates. The isolates differed in the intensity of hemolytic zone surrounding the colonies. However, most of them showed a strong β hemolysis (78.18%). The remaining isolates had a moderate zone of β hemolysis on the blood agar. Fifty isolates (72.7%) exhibited strong hemolysis enhanced by *Staphylococcus aureus* (ATCC 29,213) in CAMP test. The rest of isolates had a weak CAMP reaction. Smears from cultured colonies also contained irregular Gram‐positive coccobacilli. Catalase, oxidase, motility, urease, nitrate reduction, and esculin tests were negative for all isolates. On the contrary, the results of gelatin and Loeffler serum hydrolysis were positive. In litmus milk, production of acid, clot, reduction, and protein digestion were observed. Results of lactose, sucrose, xylose, maltose, and mannitol fermentation and alkaline phosphatase test were different for individual isolates. Five different biotypes of *T*. *pyogenes* were determined based on the results from performed biochemical tests.

**TABLE 2:** Micro‐organisms isolated from mastitis in dairy cattle

Isolates	No (%)
*Trueperella Pyogenes*	12 (21.82)
*Trueperella Pyogenes*, *E. coli*	8 (14.54)
*Trueperella Pyogenes*, *Fusobacterium necrophorum*	7 (12.72)
*Trueperella Pyogenes*, *Streptococcus dysgalactiae*	7 (12.72)
*Trueperella Pyogenes*, *Staphylococcus aureus*	5 (9.09)
*Trueperella Pyogenes*, *Streptococcus agalactiea*	3 (5.45)
*Trueperella Pyogenes*, *Enterococcus faecalis*	3 (5.45)
*Trueperella Pyogenes*, *Proteus mirabilis*	2 (3.63)
*Trueperella Pyogenes*, *Streptococcus α hemolytic*	2 (3.63)
*Trueperella Pyogenes*, *Staphylococcus intermedius*	2 (3.63)
*Trueperella Pyogenes*, *Pseudomonas aeruginosa*	2 (3.63)
*Trueperella Pyogenes*, *Klebsiella*	1 (1.82)
*Trueperella pyogenes*, *Serratia marcescens*	1 (1.53)
Total	55 (100)

In this study, presence of *T*. *pyogenes* isolated from bovine mastitis infections has been identified by biochemical test and molecular diagnostic method including detection of gene encoding virulence factors (VFs) of *T*. *pyogenes*. However, detection of gene encoding VFs is very important for describing and investigation of pathogenesis in clinical infection diseases (Rzewuska et al., 2012). Whereas some phenotypic characteristics of this bacteria identified by biochemical tests have been changed between generations, molecular diagnosis methods of gene encoding these properties are described more practical and precise for identification of this pathogen in clinical detections (Al‐Tarazi et al., 2012). In the present study, according to phenotypic and biochemical analysis, it has been demonstrated that hemolysis, CAMP test, and some biochemical characteristics were varied between isolated pathogens which have been categorized into five separate biotypes. The isolated included in biotypes 1 and 3 were significantly observed complete positive in CAMP and hemolysis tests with more severe clinical symptoms. Biotype study of these pathogens should be helpful to have better understanding of pathogenesis and diseases controlling with higher performance.

According to the data from Table 2, individual infection by *T*. *pyogenesis* and coinfection of this pathogen and other micro‐organisms had an essential role in causing bovine mastitis. Competitive conditions including obtaining nutrients, colonization, and releasing metabolites are key factors in the severity of caused coinfection and the viability of these pathogens (Tamai et al., 2018). It has been demonstrated in our study that coinfection caused by *T*. *pyogenesis* and other pathogens such as *E*. *coli*, *S*. *dysgalactiae*, and *Fusobacterium necrofurom* are usually included in biotype groups 1 and 3, associated with summer season with higher severity clinical symptoms. It is demonstrated that some critical factors at the farm such as control of mosquitos and physical injuries of teat during milking operation lead to observing more *T*. *pyogenesis* coinfection with other mastitis bacteria.

### 3.2 Antimicrobial susceptibility of the isolates

The majority of *T*. *pyogenes* isolates were highly susceptible to the amoxicillin, ampicillin, gentamicin, ceftriaxon, chloramphenicol (100%), and ceftiofur (98.2%) used in this study. High resistance rate was observed against trimethoprim sulfamethoxazole (63.65%), erythromycin (34.6%), tylosin (34.6%), and tetracycline (34.5%). Complete antimicrobial susceptibility testing results are shown in Table 3.

**TABLE 3:** The results of antibiotic susceptibility test of *T*. *pyogenes* isolated from mastitis samples

Antibiotic	S	I	R	Antibiotic	S	I	R
PG	100%	0%	0%	CEF	98.2%	1.8%	0%
AP	100%	0%	0%	CFM	85.4%	5.4%	9.2%
A	100%	0%	0%	MN	83.6%	7.3%	9.1%
T	42.5%	23%	34.5%	CRO	100%	0%	0%
E	56.3%	9.1%	34.6%	CFX	94.5%	1.8%	3.7%
TY	60%	5.4%	34.6%	CPM	87.3%	9.1%	3.6%
ENF	72.7%	5.4%	21.9%	LEV	87.3%	7.3%	5.4%
CIP	74.5%	9.1%	16.4%	S	63.5%	11%	25.5%
GM	100%	0%	0%	RP	100%	0%	0%
SPC	83.6%	9.1%	7.3%	ATH	30.9%	5.45%	63.65%
L	76.3%	9.1%	14.7%	TS	26.7%	0%	72.3%
C	100%	0%	0%				

Abbreviations: I, Intermediate; R, Resistance; S, susceptible.

Recently, the multidrug resistance properties of pathogens have been introduced as a critical problem in the world (Lim et al., 2014). Overusing of antibiotherapy, uncompleted treatment period, inappropriate drug prescription, horizontal antimicrobial resistance gene transferring, and using antibiotics as growth promoters are the key factors of multidrug susceptibility (Doyle, 2015). In our study, we demonstrated that the single or multidrug susceptibility of *T*. *pyogenesis* concerns as it was reported for other pathogens previously. The isolates detected in this study were observed principally resistant to trimethoprim–sulfamethoxazole, erythromycin, and streptomycin; and susceptible to penicillin, amoxicillin, gentamicin, ampicillin, rifampicin, ceftriaxone, and chloramphenicol. Use of antibiotics, to which this pathogen was susceptible, is suggested for treatment of mastitis and infection caused by *T*. *pyogenesis*. Development of lipogranulomatouse, losing of antibiotic effects on granulomas, and causing simultaneous coinfection by facultative and obligate anaerobe pathogens lead to hard treat in care of mastitis at higher severity level; however, the delay in diagnosis of the disease and inappropriate drug prescription weakened the probability of effective treatment (Zhang, Zhao, et al., 2017). The presence of *tetW* gene and macrolide antibiotic resistance ones including *ermB* and *ermX* were demonstrated between 58% and 34.5% of isolates, respectively, which showed the importance of horizontal multidrug resistance gene transferring between bacteria. 94.7% of isolates in this study with tylosin resistance characteristic presented *ermX* gene corresponded with the result reported by Zastempowska and Lassa (2012). Also, they found *tetW*, *ermX*, and *ermB* genes in the *T*. *pyogenesis* isolates that identified between cows with clinical mastitis symptoms.

### 3.3 Virulence factors of the isolates

DNA fragment of the expected size (122 bp) was detected in all isolates from summer mastitis samples. The 16S‐23S rDNA sequence of the isolated bacterial isolate was compared. All of the isolates were tested for 8 genes encoding virulence factors by PCR. The genes *plo*, *fimA*, *nanP*, and *nanH* were identified in all isolates. Genes *fimC*, *fimE*, and *fimG* were detected in 42 (76.36%), 42 (76.36%), and 46 (83.63%) isolates, respectively. The least frequent virulence gene in *T. pyogenes* isolates was *cbpA* with a rate of 72.72%. Fifty‐five isolates were distributed in 8 genotype groups (Table 4). The isolates placed in the most frequent genotype group possessed all 8 genes encoding virulence factors. In contrast, the least frequent genotype was detected in 4 isolates which only carried *fimA*, *plo*, *nanH*, *and nanP* genes. Tetracycline resistance gene *tetW* and macrolide resistance genes *ermB* and *ermX* were detected in 32 (58.18%), 22 (40%), and 29 (52.72%) isolates, respectively.

**TABLE 4:** Virulotypes of *T*. *pyogenes* isolates from mastitis in dairy cattle

Genotype	No. of isolates	%
*Plo*, *nanH*, *nanP*, *fimA*, *fimC*, *fimE*, *fimG*, *cbpA*	36	65.5
*Plo*, *nanH*, *nanP*, *fimA*, *fimC*, *fimE*, *cbpA*	2	3.6
*Plo*, *nanH*, *nanP*, *fimA*, *fimE*, *fimG*, *cbpA*	2	3.6
*Plo*, *nanH*, *nanP*, *fimA*, *fimC*, *fimG*	3	5.5
*Plo*, *nanH*, *nanP*, *fimA*, *fimE*	2	3.6
*Plo*, *nanH*, *nanP*, *fimA*, *fimG*	5	9.1
*Plo*, *nanH*, *nanP*, *fimA*, *fimC*	1	1.8
*Plo*, *nanH*, *nanP*, *fimA*	4	7.3
Total	55	100

Presenting of putative and known virulence genes are very determining in pathogenicity of the *T*. *pyogenesis* isolates. Virulence genes including *plo*, *nanH*, *nanP*, and *fimA* were detected in all isolates identified in the present study. They found only *plo* and *fimA* genes as virulence factors in all isolates; however, other virulence genes were detected with varied frequencies. Presence of each 8 virulence factor genes in 65.5% of isolates indicated the importance of identification of these genes in determining the pathogenicity of this pathogen (Zastempowska & Lassa, 2012). Alkasir et al. in the year 2016 also detected all virulence genes consisting of *plo*, *nanH*, *nanP*, and *cpbA* in all *T*. *pyogenesis* isolates identified in animals with bovine mastitis symptoms. Expression of these virulence genes indicated an important role in pathogenicity and biofilm formation of *T*. *pyogenesis* isolates (Alkasir et al., 2016). With investigation of association between presence of virulence factor genes and some phenotypic properties of isolates, it is demonstrated that the isolates with complete hemolysis, positive CAMP test, and detected common virulence genes lead to disease with higher severity of clinical symptoms. Also; in this study, the isolates identified during summer season were presented the virulence factor genes with higher frequencies in comparison with other times as the most of these identified isolates were categorized in biotype 1 and 3 groups.

### 3.4 Clonal relationship among the isolates

All 55 *T*. *pyogenes* isolates and the standard strain (ATCC 19,411) were discriminated in 7 main groups, including groups A, B, C, D, E, F, and G (Figure 3). The majority of isolates belonged to group A with 7 clonal types and 31 isolates. Group B included with 2 clonal types and 9 isolates, group C one clonal type and 3 isolates, group D one clonal type and 2 isolates, group E one clonal type and one isolate, and group F included with 2 clonal types and 9 isolates, respectively. The reference strain did not match with any isolate type and was categorized as type G. Among all 14 types, types IV, II, and XIII were the predominant ones with 8 and 9 isolates, respectively, while types III and XIII (each with one isolate) were the least common types (Figure 3). Data regarding the relationship between clonal types, virulence factor genes, biochemical profile, CAMP test results, and the sampling time are summarized in (Table 5).

**TABLE 5:** Relationship between clonal types, virulence factor genes, antibiotic resistance genes of isolates, biochemical profiles, CAMP results, and sampling time of the isolates

Box‐PCR type	No. of isolates	Virulence factor genes								Biochemical patterns								CAMP test	
		*plo*	*nanP*	*nanH*	*fimA*	*fimC*	*fimG*	*fimE*	*cbp*	*tetW*	*ermX*	*ermB*	1	2	3	4	5	S	W
I	4	4	4	4	4	2	3	3	2	3	3	3	1	0	0	2	1	2	2
II	7	7	7	7	7	5	3	6	5	4	4	3	2	3	1	1	0	4	3
III	1	1	1	1	1	1	1	1	1	1	1	0	1	0	0	0	0	1	0
IV	8	8	8	8	8	5	7	4	4	4	4	2	0	1	1	4	2	5	3
V	4	4	4	4	4	4	4	4	4	2	1	1	1	0	2	0	1	4	0
VI	5	5	5	5	5	5	5	5	5	2	1	0	3	0	1	0	1	5	0
VII	2	2	2	2	2	2	2	2	2	0	1	0	0	0	2	0	0	2	0
VIII	4	4	4	4	4	4	4	4	4	1	1	0	2	0	2	0	0	4	0
IX	5	5	5	5	5	5	5	5	5	3	3	3	1	0	2	0	2	5	0
X	3	3	3	3	3	0	1	0	0	2	1	2	0	3	0	0	0	0	3
XI	2	2	2	2	2	2	2	2	2	1	1	1	1	0	1	0	0	2	0
XII	1	1	1	1	1	1	1	1	1	1	1	1	1	0	0	0	0	1	0
XIII	7	7	7	7	7	4	6	3	3	6	5	5	2	4	0	0	1	3	4
XIV	2	2	2	2	2	2	2	2	2	2	2	1	2	0	0	0	0	2	0
Total	55	55	55	55	55	42	46	42	40	32	29	22	17	11	12	7	8	40	15

BOX‐PCR is considered as a suitable universal fingerprinting technique for molecular typing of bacteria today (Masco et al., 2003). By this method, isolates caused mastitis at the present study have been identified and categorized into 7 different clonal groups, whereas high significance correlation was observed between presented virulence genes, biochemical, and phenotypical properties of some clonal groups. All 8 virulence genes, positive hemolysis and CAMP tests, and strong clinical symptoms were observed in some isolates of *T*. *pyogenesis* in this study; however, they were indifferent sampling areas, same clonal related type, and included principally in biotypes 1 and 3. More antibiotic susceptibility genes were observed in some clonally related types; however, in some other ones with different biotype categorization, less virulence genes were detected. Zhao et al. in the year 2013 isolated *T*. *pyogenesis* from seeking forest musk deer. According to the obtained result from BOX‐PCR typing, they found 3 related clonal types by the reference line at 0.73 (Zhao et al., 2013); however, isolates in this study were categorized and separated into 7 different groups significantly by the reference line at 0.8 generated automatically. Also, Silva et al., (2008) reported the same result as we obtained in this study. They isolated *T*. *pyogenesis* from dairy cows with clinical metritis and determined 10 clonal types by BOX‐PCR technique with the similarity cutoff at 84%. They evaluated the relationship between virulence factor genes and clonally related types as it has been done in this study for *T*. *pyogenesis* isolated from dairy cows with clinical mastitis symptoms (Silva et al., 2008). More sample size and studies for investigation of different biotype and clonally related types are suggested for controlling this pathogen.

## 4 CONCLUSION

As concluded in this study, the highest percentage (55%) of dairy cows with clinical mastitis symptoms was caused by *T*. *pyogenesis* which indicated the importance of this pathogen contributing to clinical mastitis in dairy cows. Also, a significant correlation was observed between presence of virulence factor genes of isolated pathogen, the utter infected types, and biochemical patterns. Isolated *T*. *pyogenesis* showed multidrug resistance indicated the prudent use of antimicrobials is needed in treatment of diseases caused by this pathogen. Isolates were categorized into 7 different clonal related types by COX‐PCR with significance relationship to clonal types, biochemical profile, CAMP test result, and sampling time. Further investigations with higher sample size in different sampling time and areas are suggested for more precise study of this pathogen.

## CONFLICT OF INTEREST

All authors declare that they have no conflict of interest.

## AUTHOR CONTRIBUTIONS

**Iradj Ashrafi Tamai:** Conceptualization (equal); Methodology (equal); Writing‐original draft (equal). **Abdolmajid Mohammadzadeh:** Data curation (equal); Project administration (equal); Supervision (equal). **Taghi Zahraei Salehi:** Resources (equal); Visualization (equal). **Pezhman Mahmoodi:** Formal analysis (equal); Funding acquisition (equal). **Babak Pakbin:** Writing‐original draft (equal); Writing‐review & editing (equal).

## Data Availability

All authors confirm that the data supporting the findings of this study are available within the article.

## References

[fsn32431-bib-0001] Alkasir, R., Wang, J., Gao, J., Ali, T., Zhang, L., Szenci, O., Bajcsy, Á. C., & Han, B. O. (2016). Properties and antimicrobial susceptibility of Trueperella pyogenes isolated from bovine mastitis in China. Acta Veterinaria Hungarica, 64(1), 1–12. 10.1556/004.2016.001 26919137

[fsn32431-bib-0002] Al‐Tarazi, Y., Hijazin, M., Alber, J., Lämmler, C., Hassan, A. A., Timke, M., & Zschöck, M. (2012). Phenotypic and genotypic characteristics of Trueperella (Arcanobacterium) pyogenes isolated from lung abscesses of one‐humped camels (Camelus dromedarius) in Jordan. Journal of Camelid Science, 5, 99–104.

[fsn32431-bib-0003] Bicalho, M., Machado, V., Oikonomou, G., Gilbert, R., & Bicalho, R. (2012). Association between virulence factors of Escherichia coli, Fusobacterium necrophorum, and Arcanobacterium pyogenes and uterine diseases of dairy cows. Veterinary Microbiology, 157(1–2), 125–131. 10.1016/j.vetmic.2011.11.034 22186615

[fsn32431-bib-0004] Bogni, C., Odierno, L., Raspanti, C., Giraudo, J., Larriestra, A., Reinoso, E., & Frigerio, C. (2017). War against mastitis: Current concepts on controlling bovine mastitis pathogens. Revista Argentina De Microbiología, 10, 2.

[fsn32431-bib-0005] Doyle, M. E. (2015). Multidrug‐resistant pathogens in the food supply. Foodborne Pathogens and Disease, 12(4), 261–279. 10.1089/fpd.2014.1865 25621383

[fsn32431-bib-0006] Hadimli, H. H., & Kav, K. (2011). The molecular characterization of Arcanobacterium pyogenes strains isolated from samples of sheep and cattle. Kafkas Universitesi Veteriner Fakultesi Dergisi, 17(6), 893–899.

[fsn32431-bib-0007] Lim, C. J., Cheng, A. C., Kennon, J., Spelman, D., Hale, D., Melican, G., Sidjabat, H. E., Paterson, D. L., Kong, D. C. M., & Peleg, A. Y. (2014). Prevalence of multidrug‐resistant organisms and risk factors for carriage in long‐term care facilities: A nested case–control study. Journal of Antimicrobial Chemotherapy, 69(7), 1972–1980. 10.1093/jac/dku077 24710025

[fsn32431-bib-0008] Machado, V. S., Bicalho, M. L. D. S., Meira Junior, E. B. D. S., Rossi, R., Ribeiro, B. L., Lima, S., Santos, T., Kussler, A., Foditsch, C., Ganda, E. K., Oikonomou, G., Cheong, S. H., Gilbert, R. O., & Bicalho, R. C. (2014). Subcutaneous immunization with inactivated bacterial components and purified protein of Escherichia coli, Fusobacterium necrophorum and Trueperella pyogenes prevents puerperal metritis in Holstein dairy cows. PLoS One, 9(3), e91734. 10.1371/journal.pone.0091734 24638139 PMC3956715

[fsn32431-bib-0009] Machado, V. S., & Bicalho, R. C. (2014). Complete genome sequence of Trueperella pyogenes, an important opportunistic pathogen of livestock. Genome Announcements, 2(2), e00400–00414. 10.1128/genomeA.00400-14 24786956 PMC4007991

[fsn32431-bib-0010] Malinowski, E., Lassa, H., Markiewicz, H., Kaptur, M., Nadolny, M., Niewitecki, W., & Ziętara, J. (2011). Sensitivity to antibiotics of Arcanobacterium pyogenes and Escherichia coli from the uteri of cows with metritis/endometritis. The Veterinary Journal, 187(2), 234–238. 10.1016/j.tvjl.2009.12.010 20129803

[fsn32431-bib-0011] Masco, L., Huys, G., Gevers, D., Verbrugghen, L., & Swings, J. (2003). Identification of Bifidobacterium species using rep‐PCR fingerprinting. Systematic and Applied Microbiology, 26(4), 557–563. 10.1078/072320203770865864 14666984

[fsn32431-bib-0012] Pohl, A., Lübke‐Becker, A., & Heuwieser, W. (2018). Minimum inhibitory concentrations of frequently used antibiotics against Escherichia coli and Trueperella pyogenes isolated from uteri of postpartum dairy cows. Journal of Dairy Science, 101(2), 1355–1364. 10.3168/jds.2017-12694 29153524

[fsn32431-bib-0013] Pomeroy, B., Sipka, A., Hussen, J., Eger, M., Schukken, Y., & Schuberth, H.‐J. (2017). Counts of bovine monocyte subsets prior to calving are predictive for postpartum occurrence of mastitis and metritis. Veterinary Research, 48(1), 13. 10.1186/s13567-017-0415-8 28222802 PMC5320682

[fsn32431-bib-0014] Ribeiro, M., Risseti, R., Bolaños, C., Caffaro, K., de Morais, A. , Lara, G., & Franco, M. (2015). Trueperella pyogenes multispecies infections in domestic animals: A retrospective study of 144 cases (2002 to 2012). Veterinary Quarterly, 35(2), 82–87. 10.1080/01652176.2015.1022667 25793626

[fsn32431-bib-0015] Rzewuska, M., Stefańska, I., Osińska, B., Kizerwetter‐Świda, M., Chrobak, D., Kaba, J., & Bielecki, W. (2012). Phenotypic characteristics and virulence genotypes of Trueperella (Arcanobacterium) pyogenes strains isolated from European bison (Bison bonasus). Veterinary Microbiology, 160(1–2), 69–76. 10.1016/j.vetmic.2012.05.004 22658663

[fsn32431-bib-0016] Silva, E., Gaivão, M., Leitão, S., Jost, B. H., Carneiro, C., Vilela, C. L., Lopes da Costa, L., & Mateus, L. (2008). Genomic characterization of Arcanobacterium pyogenes isolates recovered from the uterus of dairy cows with normal puerperium or clinical metritis. Veterinary Microbiology, 132(1–2), 111–118. 10.1016/j.vetmic.2008.04.033 18547748

[fsn32431-bib-0017] Srednik, M. E., Usongo, V., Lépine, S., Janvier, X., Archambault, M., & Gentilini, E. R. (2018). Characterisation of Staphylococcus aureus strains isolated from mastitis bovine milk in Argentina. Journal of Dairy Research, 85(1), 57–63. 10.1017/S0022029917000851 29468991

[fsn32431-bib-0018] Tamai, I. A., Mohammadzadeh, A., Salehi, T. Z., & Mahmoodi, P. (2018). Genomic characterisation, detection of genes encoding virulence factors and evaluation of antibiotic resistance of Trueperella pyogenes isolated from cattle with clinical metritis. Antonie Van Leeuwenhoek, 14, 1–13. 10.1007/s10482-018-1133-6 30066209

[fsn32431-bib-0019] Ülbegi‐Mohyla, H., Hijazin, M., Alber, J., Lämmler, C., Hassan, A., Abdulmawjood, A., & Zschöck, M. (2010). Identification of Arcanobacterium pyogenes isolated by post mortem examinations of a bearded dragon and a gecko by phenotypic and genotypic properties. Journal of Veterinary Science, 11(3), 265–267. 20706035 10.4142/jvs.2010.11.3.265 PMC2924489

[fsn32431-bib-0020] Weinstein, M., Patel, J., & Bobenchik, A. (2019). Clinical and laboratory standards institute. Performance Standards for Antimicrobial Susceptibility Testing, 88–89.

[fsn32431-bib-0021] Zastempowska, E., & Lassa, H. (2012). Genotypic characterization and evaluation of an antibiotic resistance of Trueperella pyogenes (Arcanobacterium pyogenes) isolated from milk of dairy cows with clinical mastitis. Veterinary Microbiology, 161(1–2), 153–158. 10.1016/j.vetmic.2012.07.018 22868181

[fsn32431-bib-0022] Zhang, D., Zhao, J., Wang, Q., Liu, Y., Tian, C., Zhao, Y., Yu, L., & Liu, M. (2017). Trueperella pyogenes isolated from dairy cows with endometritis in Inner Mongolia, China: Tetracycline susceptibility and tetracycline‐resistance gene distribution. Microbial Pathogenesis, 105, 51–56. 10.1016/j.micpath.2017.02.010 28188901

[fsn32431-bib-0023] Zhang, W., Wang, P., Wang, B., Ma, B., & Wang, J. (2017). A combined Clostridium perfringens/Trueperella pyogenes inactivated vaccine induces complete immunoprotection in a mouse model. Biologicals, 47, 1–10. 10.1016/j.biologicals.2017.04.002 28427828

[fsn32431-bib-0024] Zhao, K., Tian, Y., Yue, B., Wang, H., & Zhang, X. (2013). Virulence determinants and biofilm production among Trueperella pyogenes recovered from abscesses of captive forest musk deer. Archives of Microbiology, 195(3), 203–209. 10.1007/s00203-013-0869-7 23354327

